# Association of triglyceride-glucose index trajectory and frailty in urban older residents: evidence from the 10-year follow-up in a cohort study

**DOI:** 10.1186/s12933-023-02002-3

**Published:** 2023-09-29

**Authors:** Yin Yuan, Simiao Chen, Chunjin Lin, Xiaoming Huang, Siyang Lin, Feng Huang, Pengli Zhu

**Affiliations:** 1 Department of Geriatric Medicine, Fujian Provincial Hospital, Fuzhou, China; 2 Shengli Clinical Medical College, Fujian Medical University, Fuzhou, China; 3 Fujian Provincial Institute of Clinical Geriatrics, Fuzhou, China; 4 Fujian Provincial Center of Geriatrics, Fuzhou, China; 5 Fujian Provincial Key Laboratory of Geriatrics, Fuzhou, China; 6 Xiamen Medical College, Xiamen, China

**Keywords:** Triglyceride-glucose index, Insulin resistance, Frail older adults

## Abstract

**Background:**

Frailty is an age-related geriatric syndrome that leads to a series of clinically negative events. A better understanding of the factors associated with frailty assists in preventing its progression. The triglyceride-glucose (TyG) index, a simple alternative index of insulin resistance, has not yet been proven to be associated with frailty. The present study aimed to investigate the association between the TyG index and its trajectory with frailty from a cross-sectional, retrospective and prospective level based on an ongoing cohort.

**Methods:**

This longitudinal study included 1,866 older residents from the “Fujian prospective aging cohort” (ChiCTR 2,000,032,949). The TyG index was calculated as ln [fasting triglyceride (mg/dL) ╳ fasting plasma glucose (mg/dL)/2] and group-based trajectory model (GBTM) was applied to identify the trajectory of TyG index. The association between different trajectory groups of TyG index with frailty risk were estimated using multinomial logistic regression analysis.

**Results:**

In the cross-sectional analysis, the highest quartile of the TyG index was associated with an increased risk of frailty (TyG index Q4 vs. Q1, OR = 1.50, 95% CI 1.00–2.25, *P* = 0.048). Restricted cubic splines demonstrated an increasing trend for TyG index and frailty risk. During a follow-up of ten years, three distinct trajectories of the TyG index were identified: low-stable (n = 697, 38.3%), moderate-stable (n = 910, 50.0%) and high-stable (n = 214, 11.7%). Compared with those in the stable-low group of TyG index trajectory, the ORs (95% CI) of prefrailty and frailty risk were 1.79 (95% CI 1.11–2.88) and 2.17 (95% CI 1.01–3.88) for the high-stable group, respectively (*P* = 0.017 and *P* = 0.038). In the subgroup analysis, the association of the high-stable trajectory of TyG and frailty status were only observed in subjects with BMI ≥ 24 kg/m^2^. Prospectively, the highest quartile of the TyG index was associated with a 2.09-fold significantly increased risk of one-year ADL/IADL decline (*P* = 0.045).

**Conclusions:**

The present study suggests a potential role for a high and sustainable level of TyG index in the risk of frailty. The trajectories of the TyG index can help to identify older individuals at a higher risk of frailty who deserve primitive preventive and therapeutic approaches.

**Supplementary Information:**

The online version contains supplementary material available at 10.1186/s12933-023-02002-3.

## Introduction

Frailty is a dynamic geriatric syndrome characterized by the decline of physical homeostatic reserve and increased vulnerability to external stress [1]. Frailty increases the risk of cardiovascular diseases, mortality and the need for hospitalization and institutional care, also reduces the net benefits of necessary medical interventions due to competing risks [2]. Frailty develops relatively slowly, especially in the early stages, so as to obtain insufficient attention. Considering the detrimental impact of functional decline brought by frailty, the early identification of frailty status and the prevention of its progression would be crucial. One feasible approach is to identify potential risk factors or novel biomarkers of frailty. The physiopathology of frailty is complex and multifactorial. It has been shown that chronic low-grade inflammation and oxidative stress play critical roles [3].

Previous studies have suggested that chronic inflammation leads to insulin resistant (IR) and muscle dysfunction, subsequently results in the worsening of the frailty state [4, 5]. IR impairs the ability of muscles to process glucose and the muscle strength is weakened thereafter [6]. The triglyceride-glucose (TyG) index, conveniently calculated by triglyceride and fasting glucose levels, has been regarded as a good alternative surrogate marker for the established parameters of IR [7]. Accumulating evidence indicated that the TyG index is associated with cardiovascular and metabolic risk, predominantly cardiovascular disease [8–11], type 2 diabetes mellitus [12], stroke [13, 14], arterial stiffness [11, 15, 16], vascular damage [17, 18] and even death [19]. Recently, a few studies reported that a higher TyG index was also associated with an increased risk of sarcopenia or low muscle mass in older populations [20–22]. Zheng et al. also claimed that handgrip strength per weight was inversely associated with TyG index in the elderly population [23]. These results suggest that TyG index could be an indicator of physical function decline. However, the previous studies were inherently limited by short follow-up durations or cross-sectional designs, with few considerations given to the variability or reversibility of the potential trajectories of TyG index. For a set of time-varying dynamic data, the group-based trajectory model (GBTM) could capture patterns that might exist on the timing, direction and extent of variation, which fills the gap left by one single measurement ignoring the differences in development, thus contributing to a clear grasp on the full course [24].

No prior research has reported the relationship of TyG index and its trajectory with the frailty status in an older population. This study was designed to investigate the association of TyG index and its trajectory with frailty based on multiple measurements over a 10-year follow-up from cross-sectional, retrospective and prospective level. The results were expected to clarify the relationship between TyG index and frailty status, and stress the importance of long-term blood lipid and glucose control in older populations.

## Methods

### Study design and participants

**Fig. 1:**
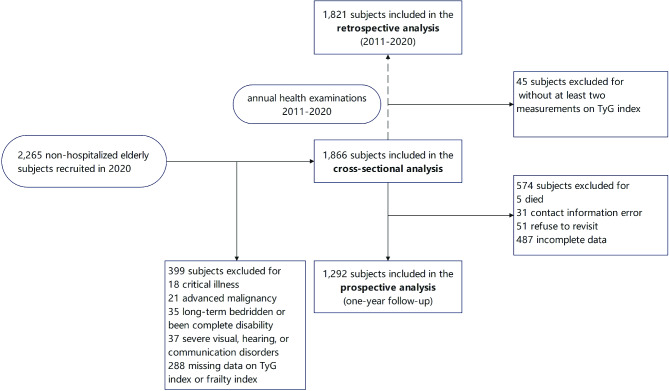
Flowchart of the study

The participants were derived from the “Fujian prospective aging cohort”, an ongoing prospective cohort established in 2020 to investigate health status based on comprehensive geriatric assessment and cardiovascular events in the non-hospitalized older population (ChiCTR 2,000,032,949), which has been described in detail previously [25]. Briefly, 2,265 subjects aged 60 years and above from Wenquan Community, Fuzhou City in May 2020 were enrolled. Subjects who had less than six months of life expectancy due to advanced malignancy or critical medical conditions, or were unable to complete the questionnaire investigation and physical examination, or who had missing data on the TyG index were excluded. Finally, 1,866 subjects were included in the cross-sectional analysis. Among these participants, based on the digital data of annual health examinations, we included 1,821 participants who took part in at least two surveys from 2011 to 2020 in the retrospective analysis. In the prospective level, subjects were invited to participate in follow-up investigations. Basic/instrumental activities of daily living (ADL/IADL), exercise habits, grip strength, gait speed, physical examination, and biochemical indicators were evaluated annually. The second visit was conducted in July 2021, which 1,292 subjects completed. Figure 1 displays the flowchart of the study. This study was approved by the Ethics Committee of Fujian Provincial Hospital (No. K2020-05-008) and was conducted according to the principles of the Declaration of Helsinki. Written informed consent was obtained from all participants.

### Measurements

#### Covariates and definitions

The following covariates were obtained from questionnaires conducted by trained interviewers: demographic characteristics (age, gender, education, living condition and monthly income), lifestyle factors (tobacco and alcohol use, exercise habits) and clinical indicators (physician-diagnosed medical conditions, the use of regular medications). Basic/instrumental activities of daily living (ADL/IADL) were evaluated by the Katz scale [26] and the Lawton instrumental activities of daily living scale [27]. Balance capacity was assessed with the timed up and go test [28]. Nutritional status was evaluated using the mini nutritional assessment-short form (MNA-SF) score [29]. Cognitive function was evaluated using the minicog scale [30]. A physical examination was performed to measure height, weight, waist circumference and office blood pressure. Mean arterial pressure (MAP) was calculated by “diastolic blood pressure + 1/3 pulse pressure difference”. Grip strength was measured by a hydraulic dynamometer (Jamar, Anaheim, CA, USA) in the sitting position. Gait speed was assessed by the 4-Metre Walk Test. Dyslipidemia was defined as having one or more of the following: fasting serum TG ≥ 1.7 mmol/l, TC ≥ 5.2 mmol/l, LDL-C ≥ 3.3 mmol/l, HDL-C < 1.0 mmol/l, or self-reported history or use of lipid-lowering medication.

### Biochemical parameters

Blood samples were collected in the morning after overnight fasting. The biochemical parameters including total cholesterol (TC), triglyceride (TG), low-density lipoprotein cholesterol (LDL-C), high-density lipoprotein cholesterol (HDL-C), fasting plasma glucose (FPG), serum albumin, serum creatinine, uric acid and hemoglobin a1c (HbA1c) were detected. TyG index was calculated using the formula: ln [fasting triglyceride (mg/dL) ╳ fasting plasma glucose (mg/dL)/2].

### Frailty index

The frailty index (FI) counts deficits in health, which is expressed as a proportion of the current deficit to the total number of deficits. According to the method recommended by Rockwood [31], this study established and constructed 40 items for the FI in accordance with the principle of selecting variables. The 40 items are presented in Supplementary Table 1. Subjects were classified as “non-frail” (FI ≤ 0.1), “pre-frail” (0.1 < FI ≤ 0.25) or frail (FI > 0.25) in order to obtain greater sensitivity for frailty risk [31–33]. In the prospective analysis, the change in the ADL/IADL score was used to illustrate the worsening of frailty state, which was calculated by the baseline ADL/IADL score (in 2020) minus the follow-up ADL/IADL score (in 2021). Participants who received positive scores were considered as “worsening of functional capacity/frailty state”, while those who received negative scores or zero were considered as “improved or stable functional capacity/frailty state”.

### Statistical analysis

Mean ± standard deviation (SD) or medians ± 25th–75th percentiles were used for presenting continuous variables. Differences between multiple groups were analyzed by ANOVA analysis or Kruskal–Wallis rank test according to the normality of the distribution. Categorical variables are reported as proportions and comparisons are performed using the chi-squared test. The association with continuous TyG index levels and frailty index was explored by multivariate linear regression. Binary logistic regression analysis was performed to calculate odds ratios (OR) with 95% confidence intervals (CI) for frailty risk (FI > 0.1 was regarded as frailty risk) according to the TyG index quartiles. Restricted cubic spline analysis was carried out to explore the dose–response association between TyG index and frailty risk. Four knots were placed at the 25th, 50th, 75th and 95th percentiles.

Group-based trajectory modeling (GBTM) was employed to identify different longitudinal TyG index trajectories during 2011–2020 [34]. A censored normal model was used for continuous outcomes. The optimal number (ranging from 2 to 5) and shape of TyG index trajectories (linear, quadratic, or cubic) were determined according to the following criteria: (1) the lowest Bayesian information criterion (BIC) and Akaike information criterion (AIC); (2) no less than 5% of the participants within each trajectory group; and (3) higher average posterior probabilities for each trajectory group (> 0.70) [11, 35]. Finally, three distinct TyG index trajectories were determined as the best-fitting model, as shown in Supplementary Tables 2, 3. Multinomial logistic regression was applied to examine the relation between the trajectory group of TyG levels and frailty risk.

Subgroup analyses were stratified by age (< 70 yrs vs. ≥70 yrs), gender (male vs. female), BMI (< 24 kg/m^2^ vs. ≥24 kg/m^2^), history of diabetes/hypertension (yes vs. no). The potential interactions between these factors and the trajectories of the TyG index were explored. Sensitivity analyses excluding participants using hypoglycemic agents or lipid-lowering medication were performed to verify the robustness of the results. All statistical analyses were conducted using the Stata/MP 17.0 (StataCorp, College Station, TX) and R (version 4.1.3) statistical software packages. A two-sided p value less than 0.05 was considered statistically significant.

## Results

### The cross-sectional relationships between TyG index and frailty

Table 1 shows the characteristics of participants according to TyG index quartiles. A total of 1,866 subjects (mean age = 71.7 years, 59.5% females) were included in the cross-sectional analysis. The FI, MNA-SF score and the prevalence of hypertension, diabetes, dyslipidemia, comorbidities and polypharmacy, as well as the levels of SBP, DBP, BMI, waist circumference, uric acid, TG, TC, LDL-C, FPG, HbA1c and albumin, exhibited an increasing trend with the elevated TyG index, except for the levels of HDL-C which showed a decreasing trend (all *P* < 0.001).

**Table 1:** Characteristics of subjects by TyG index quartiles in 2020

Variables	Total N = 1866		TyG index			*P* for trend
		Q1 (≤ 8.54) N = 472	Q2 (8.54–8.88) N = 455	Q3 (8.88–9.24) N = 483	Quartile 4 (≥ 9.24) N = 456	
**General Characteristics**						
Age (year, ‾x ± s)	71.7 ± 6.5	71.8 ± 6.4	71.5 ± 6.4	71.6 ± 6.4	72.1 ± 6.7	0.51
Female (N, %)	1111 (59.5%)	263 (55.7%)	279 (61.3%)	295 (61.1%)	274 (60.1%)	0.26
Living alone (N, %)	167 (9.0%)	32 (6.8%)	43 (9.5%)	43 (8.9%)	49 (10.7%)	0.20
Low education (N, %)	363 (19.5%)	87 (18.4%)	83 (18.2%)	90 (18.6%)	103 (22.6%)	0.28
Low income (N, %)	662 (35.7%)	187 (40.0%)	161 (35.5%)	165 (34.3%)	149 (32.7%)	0.11
**Nutrition & life style**						
Smoking (N, %)	302 (16.2%)	74 (15.7%)	67 (14.7%)	90 (18.6%)	71 (15.6%)	0.38
Drinking (N, %)	275 (14.7%)	66 (14.0%)	78 (17.1%)	64 (13.3%)	67 (14.7%)	0.37
MNA-SF score	13 (12, 14)	12 (11, 14)	13 (12, 14)	13 (12, 14)	13 (12, 14)	**< 0.001**
Regular exercise (N, %)	477 (25.6%)	111 (23.5%)	115 (25.3%)	127 (26.3%)	124 (27.2%)	0.61
**Medical condition**						
Hypertension (N, %)	1167 (62.5%)	235 (49.8%)	275 (60.4%)	321 (66.5%)	336 (73.7%)	**< 0.001**
Diabetes (N, %)	604 (32.4%)	87 (18.4%)	133 (29.2%)	151 (31.3%)	233 (51.1%)	**< 0.001**
Dyslipidemia (N, %)	586 (31.4%)	88 (18.6%)	136 (29.9%)	166 (34.4%)	196 (43.0%)	**< 0.001**
CCD (N, %)	76 (4.1%)	19 (4.0%)	16 (3.5%)	20 (4.1%)	21 (4.6%)	0.87
Comorbidity (N, %)	635 (34.0%)	113 (23.9%)	148 (32.5%)	173 (35.8%)	201 (44.1%)	**< 0.001**
Polypharmacy (N, %)	520 (27.9%)	89 (18.9%)	122 (26.8%)	146 (30.2%)	163 (35.7%)	**< 0.001**
Cognitive impairment (N, %)	287 (15.4%)	60 (12.7%)	80 (17.6%)	72 (14.9%)	75 (16.4%)	0.19
Frailty index (median, IQR)	0.13 (0.09, 0.19)	0.13 (0.08, 0.18)	0.13 (0.09, 0.18)	0.13 (0.09, 0.19)	0.14 (0.10, 0.20)	**< 0.001**
**Physical Exam & Laboratory data**						
TUG (seconds)	10.2 (9.1, 11.7)	10.2 (9.0, 11.5)	10.1 (9.1, 11.7)	10.1 (9.1, 11.6)	0.4 (9.28, 12.0)	0.13
Low grip strength (N, %)	682 (33.9%)	178 (37.7%)	137 (30.1%)	155 (32.1%)	162 (35.5%)	0.065
Slow gait speed (N, %)	207 (11.1)	41 (8.7%)	60 (13.2%)	49 (10.1%)	57 (12.5%)	0.10
SBP (mmHg)	137 (126, 149)	132 (121, 144)	136 (126, 148)	138 (129, 149)	141 (130, 154)	**< 0.001**
DBP (mmHg)	81 (74, 88)	79 (71, 86)	81 (74, 88)	81 (76, 88)	82 (75, 89)	**< 0.001**
BMI (kg/m^2^)	24.6 (22.6, 26.5)	23.4 (21.2, 25.5)	24.5 (22.6, 26.2)	24.9 (23.1, 26.9)	25.3 (23.6, 27.2)	**< 0.001**
WC (cm)	85.5 ± 8.7	82.2 ± 9.0	85.0 ± 8.5	86.7 ± 7.9	88.1 ± 8.5	**< 0.001**
Albumin (g/L)	37.1 ± 8.4	36.0 ± 8.4	36.8 ± 8.6	38.2 ± 8.0	37.3 ± 8.5	**< 0.001**
Creatinine (µmol/L)	65 (54, 79)	66 (55, 78)	64 (54, 78)	65 (54, 80)	66.5 (53.5, 80)	0.63
Uric acid (µmol/L)	365 (311, 426)	341.5 (290, 400.5)	359 (305, 423)	371 (315, 428)	393 (338, 452.5)	**< 0.001**
HDL-C (mmol/L)	1.2 (1.0, 1.4)	1.3 (1.1, 1.6)	1.2 (1.0, 1.4)	1.1 (1.0, 1.3)	1.1 (0.9, 1.2)	**< 0.001**
LDL-C (mmol/L)	2.7 (2.1, 3.4)	2.5 (1.8, 3.1)	2.8 (2.1, 3.4)	2.9 (2.2, 3.6)	2.9 (2.1, 3.5)	**< 0.001**
TC (mmol/L)	5.3 (4.5, 6.1)	5.1 (4.3, 5.7)	5.24 (4.5, 6.0)	5.5 (4.7, 6.2)	5.4 (4.6, 6.4)	**< 0.001**
TG (mmol/L)	1.4 (1.1, 2.0)	0.9 (0.8, 1.0)	1.3 (1.1, 1.4)	1.7 (1.5, 2.0)	2.5 (2.0, 3.2)	**< 0.001**
FPG (mmol/L)	5.9 (5.5, 6.1)	5.5 (5.2, 5.9)	5.9 (5.4, 6.4)	6.0 (5.5, 6.8)	7.0 (6.0, 8.9)	**< 0.001**
HbA1c (%)	5.8 (5.3, 6.4)	5.5 (5.1, 5.9)	5.7 (5.3, 6.2)	5.8 (5.4, 6.4)	6.4 (5.7, 7.4)	**< 0.001**

Q: quartiles, MNA-SF: mini-nutritional assessment-short form, CCD: cardiovascular and cerebral disease, TUG: timed up and go test, SBP: systolic blood pressure, DBP: diastolic blood pressure, BMI: body mass index, WC: waist circumference, TG: triglycerides, TC: total cholesterol, HDL-C: high-density lipoprotein cholesterol, LDL-C: low-density lipoprotein cholesterol. FPG: fasting plasma glucose, HbA1c: hemoglobin A1c. Low education was defined as the education level of primary school or below, low income as monthly income less than 3000 RMB, regular exercise as expenditure of physical activity per week < 383 kcal for men, < 270 kcal for women. Comorbidity was defined as the coexistence of ≥ 2 chronic conditions, polypharmacy as the use of ≥ 5 categories of medication. Cognitive impairment was defined as minicog score ≤ 2. Low grip strength and slow gait speed were defined according to Fried’s standard

**Fig. 2:**
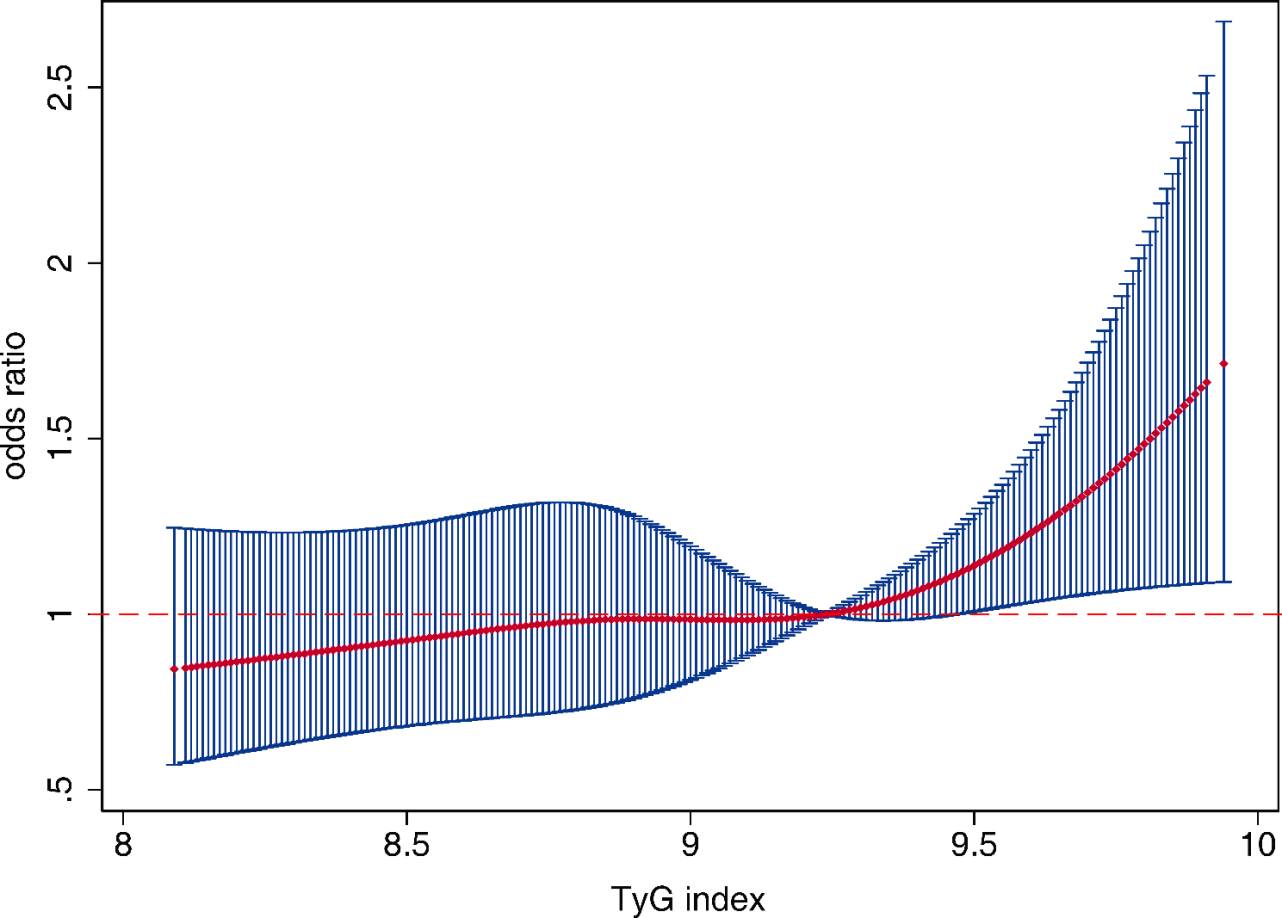
Association of TyG index levels and frailty risk. Frailty risk was defined as frailty index (FI) > 0.1. Penalized cubic spline curves based on the binary logistic regression model (adjusted by age, gender, smoking, drinking, BMI, nutritional status, exercise, chronic disease history, TC, LDL-C, HDL-C, HbA1c, MAP, use of hypoglycemic agents, and use of lipid-lowering medication). Non-linear *P* value = 0.38. Reference as TyG = 9.24.

TyG index levels were associated with FI in multivariate linear regression analysis after adjusting for covariates [Coefficient β (95% CI) = 0.014 (0.007, 0.020), *P* < 0.001], as shown in Supplementary Table 4. Multivariate binary logistic regression analysis shows that TyG index was associated with an increased frailty risk within quartiles (ORs = 1.31, 1.42, 1.84 for TyG index Q2-Q4, respectively, model 1 in Table 2). The association between the highest quartile of TyG index and frailty risk remained significant after adjustment for confounding factors (TyG index Q4 vs. Q1, ORs = 1.50, 95% CIs: 1.00-2.25, *P* = 0.048, model 3). To further explore the pattern of this association, restricted cubic splines demonstrated an increasing trend for TyG index and frailty risk (*P* for nonlinearity = 0.380). There was a slow rise in the risk of frailty until 9.24 of TyG index levels and started to increase substantially afterwards (Fig. 2).

**Table 2:** Association of TyG index with frailty risk in 2020 (n = 1866)

TyG index	Model 1		Model 2		Model 3	
	**OR (95%CI)**	***P*** **value**	**OR (95%CI)**	***P*** **value**	**OR (95%CI)**	***P*** **value**
Q1 [5.22–8.54]	Reference		Reference		Reference	
Q2 (8.54–8.88)	1.31 (1.00-1.72)	**0.047**	1.32 (0.99–1.76)	0.055	1.22 (0.88–1.69)	0.260
Q3 [8.88 ~ 9.24)	1.42 (1.08–1.87)	**0.011**	1.43 (1.08–1.89)	**0.013**	1.34 (0.95–1.88)	0.223
Q4 (9.24 ~ 12.19]	1.84 (1.38–2.45)	**< 0.001**	1.84 (1.37–2.48)	**< 0.001**	1.50 (1.00-2.25)	**0.048**

Model 1, unadjusted; Model 2, adjusted for age and gender; Model 3, further adjusted for smoking, drinking, BMI, nutritional status, exercise, chronic disease history, TC, LDL-C, HDL-C, HbA1c, MAP, use of hypoglycemic agents, and use of lipid-lowering medication

### Associations between the trajectories of TyG index and frailty risk

**Fig. 3:**
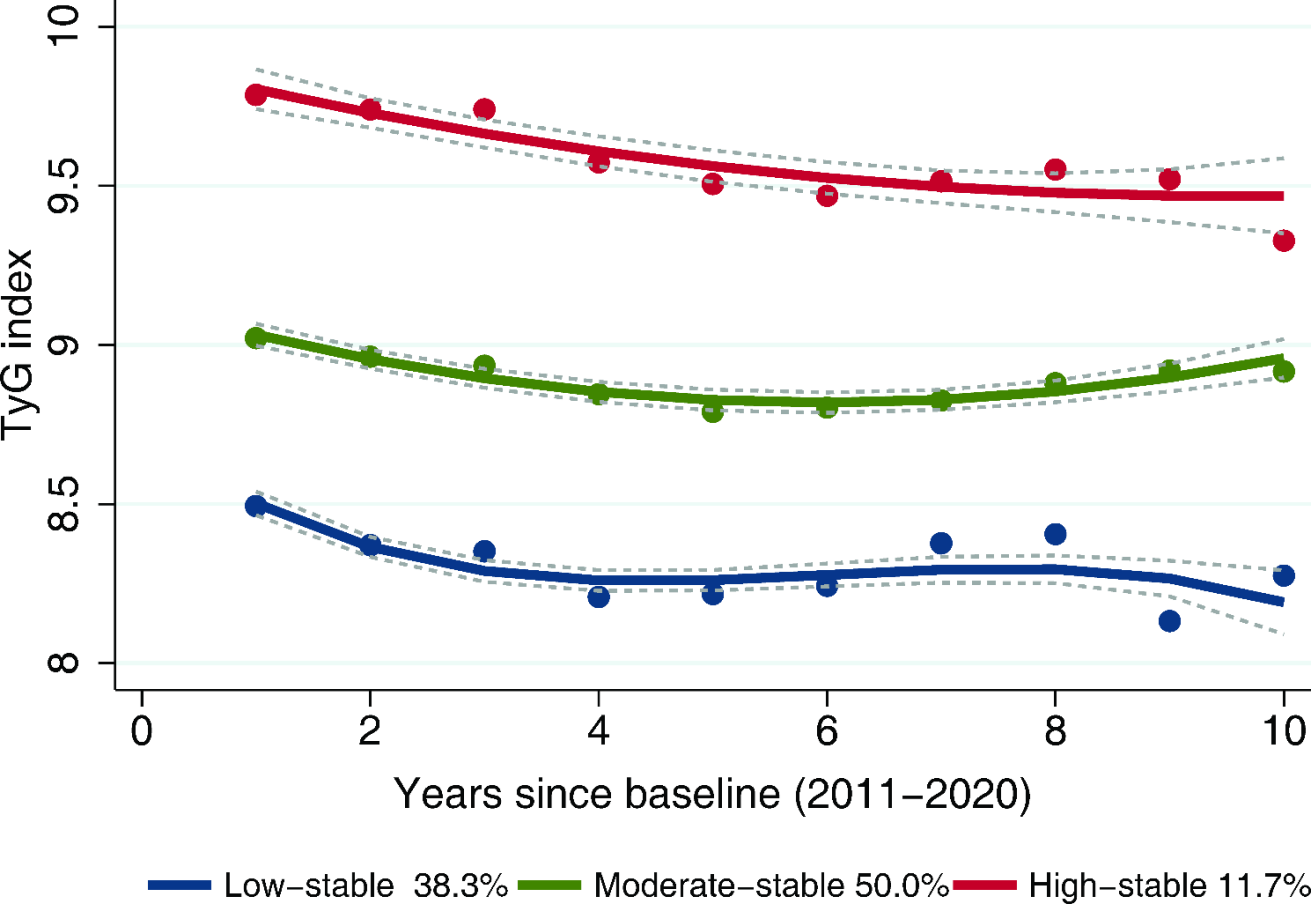
The trajectory of TyG index over a 10-year follow-up (2011–2020)

Three distinct trajectories of the TyG index from 2011 to 2020 were identified using the GBTM (Fig. 3). There were low-stable (n = 697, 38.3%), moderate-stable (n = 910, 50.0%) and high-stable (n = 214, 11.7%) groups of TyG index trajectory. As shown in Table 3, compared with subjects in the low-stable group, subjects in the moderate- and high-stable groups had a higher prevalence of hypertension, diabetes, comorbidity, polypharmacy and a higher proportion of taking antihypertensive, antidiabetic and lipid-lowering medication (all *P* < 0.001). They also had higher levels of FI, MNA-SF score, BMI, SBP, DBP, TC, TG, LDL-C, FPG, HbA1c and spent more time on the TUG test, whereas they had lower levels of HDL-C (*P* < 0.001 or *P* = 0.014). During a follow-up of ten years, multinomial logistic regression showed that in the fully adjusted model, compared with those in the low-stable group of TyG index trajectory, the risk of prefrailty and frailty being 1.79 (95% CI, 1.11–2.88) and 2.17 (95% CI, 1.01–3.88) for the subjects in the high-stable group, respectively (*P* = 0.017 and *P* = 0.038; model 2 in Table 4).

**Table 3:** Characteristics of subjects by the trajectories of TyG index from 2011 to 2020 (n = 1821)

Variables	Low-stable (N = 697)	Moderate-stable (N = 910)	High-stable (N = 214)	*P* value
**General Characteristics**				
Age, mean (SD)	71.5 ± 6.1	71.4 ± 6.3	71.3 ± 6.0	0.83
Male (%)	280 (41.4%)	382 (40.9%)	83 (39.5%)	0.89
Smoking (%)	99 (14.6%)	164 (17.6%)	32 (15.2%)	0.27
Drinking (%)	92 (13.6%)	143 (15.3%)	37 (17.6%)	0.32
Regular exercise (%)	168 (24.8%)	231 (24.7%)	63 (30.0%)	0.26
Education (primary school or below, N, %)	131 (19.4%)	173 (18.5%)	40 (19.0%)	0.91
Income (< 3000 RMB per month, N, %)	255 (37.7%)	313 (33.5%)	74 (35.2%)	0.23
**Medical history & Medication**				
MNA-SF score	12 (11, 14)	13 (12, 14)	13 (12, 14)	**< 0.001**
Hypertension (%)	342 (50.5%)	628 (67.2%)	159 (75.7%)	**< 0.001**
Diabetes (%)	126 (18.6%)	332 (35.5%)	129 (61.4%)	**< 0.001**
Comorbidities (%)	303 (44.8%)	570 (61.0%)	162 (77.1%)	**< 0.001**
Polypharmacy (%)	130 (19.2%)	274 (29.3%)	94 (44.8%)	**< 0.001**
Antihypertensive agents (%)	310 (45.8%)	574 (61.5%)	155 (73.8%)	**< 0.001**
Hypoglycemic agents (%)	86 (12.7%)	240 (25.7%)	97 (46.2%)	**< 0.001**
Lipid-lowering agents (%)	143 (21.1%)	276 (29.6%)	85 (40.5%)	**< 0.001**
Cognitive impairment (N, %)	92 (13.6%)	153 (16.4%)	21 (10.0%)	0.061
Frailty index (median, IQR)	0.12 (0.07, 0.17)	0.13 (0.09, 0.18)	0.15 (0.10, 0.20)	**< 0.001**
**Physical Exam & Laboratory data**				
TUG (seconds)	10.0 (9.0, 11.7)	10.2 (9.0, 11.5)	10.5 (9.5, 11.9)	**0.012**
Low grip strength (N, %)	242 (35.7%)	291 (31.2%)	73 (34.8%)	0.14
Slow gait speed (N, %)	71 (10.5%)	87 (9.3%)	26 (12.4%)	0.38
BMI (kg/m^2^)	24 (21.6, 25.8)	24.9 (23.1, 26.8)	25.4 (23.6, 27.4)	**< 0.001**
SBP (mmHg)	134 (123, 144)	138 (128, 149)	141.5 (131, 154)	**< 0.001**
DBP (mmHg)	80 (72, 87)	81 (75, 88)	83 (75, 91)	**< 0.001**
HDL-C (mmol/L)	1.3 (1.1, 1.5)	1.1 (1.0, 1.3)	1.1 (0.9, 1.2)	**< 0.001**
LDL-C (mmol/L)	2.7 (2.0, 3.3)	2.8 (2.2, 3.5)	2.6 (1.9, 3.3)	**< 0.001**
TC (mmol/L)	5.2 (4.4, 5.9)	5.4 (4.6, 6.2)	5.3 (4.6, 6.3)	**0.014**
TG (mmol/L)	1.0 (0.8, 1.2)	1.7 (1.3, 2.1)	3.0 (2.3, 4.0)	**< 0.001**
FPG (mmol/L)	5.6 (5.3, 6.1)	6.1 (5.6, 7)	7.1 (6.1, 9.1)	**< 0.001**
HbA1c (%)	5.6 (5.2, 5.9)	5.8 (5.4, 6.5)	6.5 (5.8, 7.5)	**< 0.001**

MNA-SF: mini-nutritional assessment-short form, BMI: body mass index, SBP: systolic blood pressure, DBP: diastolic blood pressure, HDL-C: high-density lipoprotein cholesterol, LDL-C: low-density lipoprotein cholesterol, TC: total cholesterol, TG: triglycerides, FPG: fasting plasma glucose, HbA1c: hemoglobin A1c. Comorbidity was defined as the coexistence of ≥ 2 chronic conditions, polypharmacy as the use of ≥ 5 categories of medication

**Table 4:** Frailty risk for various levels of TyG index trajectory groups

TyG index trajectories	Model 1 OR (95% CI)	*P* value	Model 2 OR (95% CI)	*P* value
**Prefrailty**				
Low-stable	Reference		Reference	
Moderate-stable	1.37 (1.08–1.74)	**0.009**	1.18 (0.90–1.54)	0.234
High-stable	2.40 (1.59–3.63)	**< 0.001**	1.79 (1.11–2.88)	**0.017**
**Frailty**				
Low-stable	Reference		Reference	
Moderate-stable	1.51 (0.97–2.36)	0.069	1.07 (0.65–1.78)	0.780
High-stable	4.48 (2.31–8.69)	**< 0.001**	2.17 (1.01–3.88)	**0.038**

Model 1: Adjusted for age, gender, smoking, drinking, BMI, nutritional status, and exercise; Model 2: adjusted for model 1 covariates plus chronic disease history, TC, LDL-C, HDL-C, MAP, HbA1c, hypoglycemic agents, and lipids-lowering medication

### Sensitivity and subgroup analysis

**Fig. 4:**
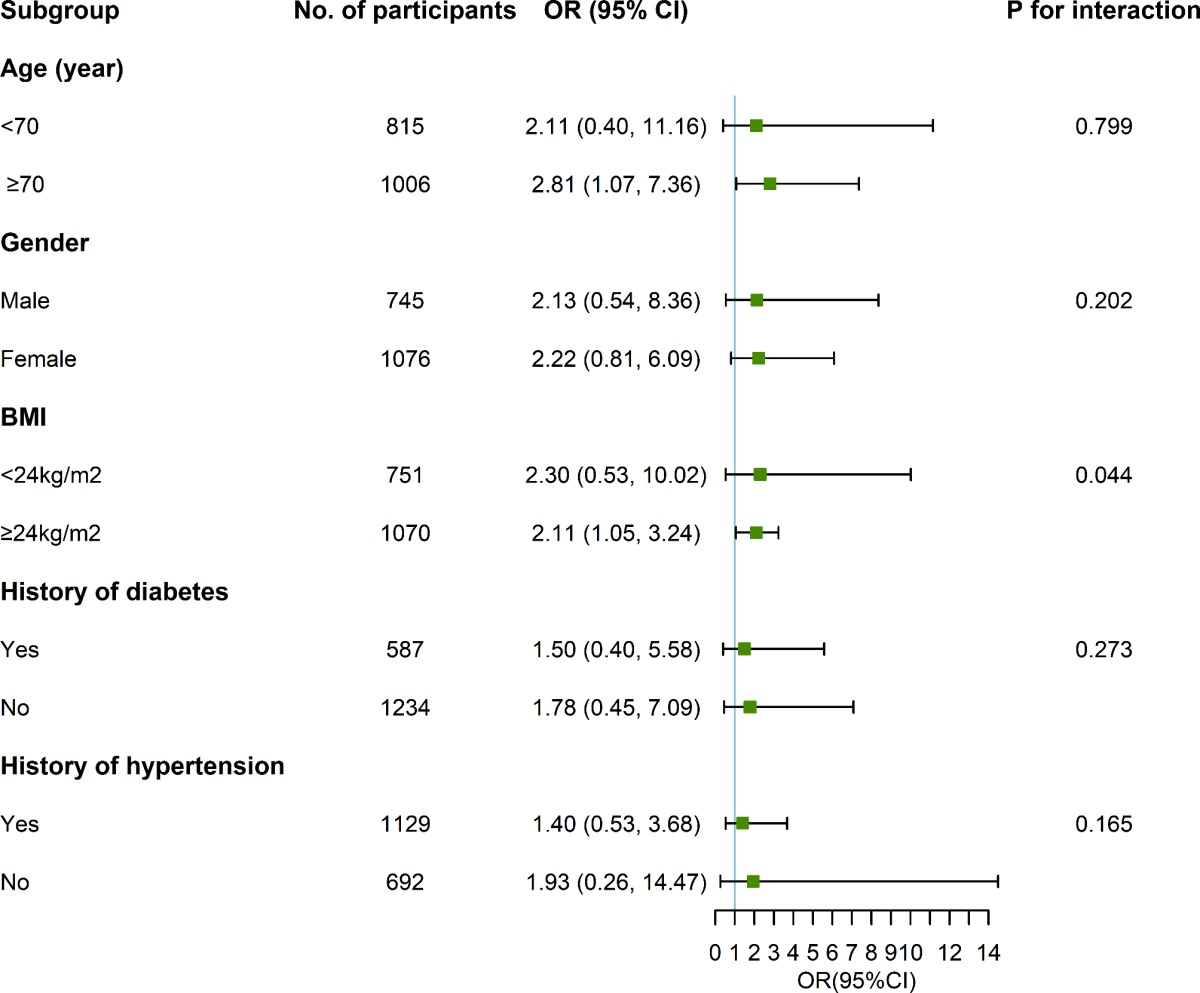
Subgroup analysis of the association with frailty risk and the stable-high trajectory of TyG index

**Fig. 5:**
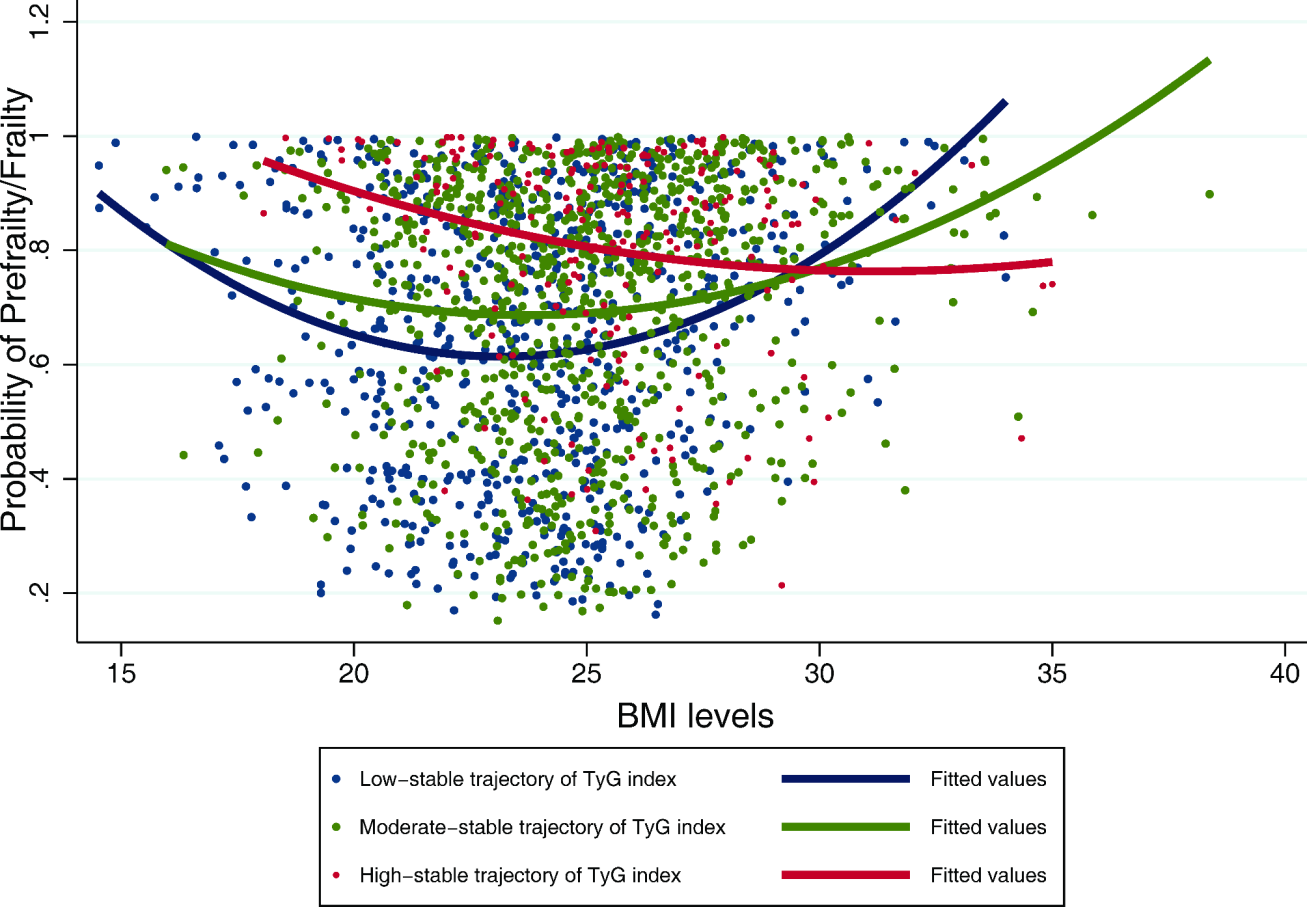
Interaction plot of the trajectories of TyG index and BMI levels

In the sensitivity analysis, excluding subjects with the use of lipid-lowering medication (n = 504) and hypoglycemic agents (n = 423), the findings were consistent with the main results after adjusting for known risk factors (Supplementary Table 5). The results of subgroup analysis were shown in Fig. 4 and Supplementary Table 6. The association between the high-stable trajectory of TyG index and prefrailty/frailty status were only observed in subjects with BMI ≥ 24 kg/m^2^ (*P* for interaction = 0.041 and 0.044, respectively). The Interaction plot of BMI levels and TyG trajectories was fit in Fig. 5, indicating that the effect of trajectories of TyG index on the risk of prefrailty or frailty was heterogenous at different levels of BMI.

### Associations between the TyG index and worsening frailty state

To further explore the prospective relationship between TyG index and the worsening of frailty state, the association between TyG index and ADL/IADL decline within one year was evaluated in Supplementary Table 7. Multivariate logistic regression analysis showed that the highest quartile of TyG index was associated with ADL/IADL decline compared to the lowest quartile of TyG index after adjusting for the covariates (TyG Q4 vs. Q1, ORs = 2.09, 95% CIs: 1.02–4.45, *P* = 0.045).

## Discussion

This is the first study to assess the association between frailty status and insulin resistance estimated by the TyG index in older urban residents. The present study investigated the association between TyG index and frailty status from cross-sectional, retrospective and prospective levels using an ongoing aging cohort dataset. Elevated TyG index and the high-stable trajectory group of the TyG index was found to be associated with a significantly increased risk of frailty, which persisted even after adjusting for potential confounders such as BMI, nutritional status, exercise and cardiovascular-metabolic factors. In the subgroup analysis, subjects with a higher BMI and who follow a high-stable trajectory of TyG index run a greater risk of developing prefrailty or frailty. Notably, these results remained robust in the sensitivity analyses that excluded subjects with the use of hypoglycemic or lipid-lowering agents, further accentuating the consistency of this association.

In our study, higher TyG index values were associated with greater frailty risk, even after adjusting for confounding factors. Although the association between TyG index and frailty has not been reported before, there has been evidence showing that the elevated TyG index was associated with low muscle mass or sarcopenia [20–23]. Losing muscle mass and strength in the elderly is the main factor responsible for frailty; therefore, our results generally echo the previous studies. Ahn et al. reported that higher TyG index values were found to be associated with an increased risk of low skeletal muscle mass index in Korean adults [20]. Other studies also showed that elevated levels of TyG index are a risk factor for reduced muscle mass or strength [21, 22], even in adolescents between the ages of 12 and 18 [36]. In addition to evidence from cross-sectional studies, post hoc analyses of a prospective study suggested that baseline lower handgrip strength levels were inversely associated with the three-year follow-up TyG index values, which indicate that handgrip strength can be a predictor of future insulin resistance [23]. However, in contrast to our findings, Hu et al. claimed that TyG index was associated with a reduced risk of low muscle mass in subjects with type 2 diabetes mellitus [37]. The discordance might be partially due to the fact that T2DM subjects in the low muscle mass group showed significantly decreased TG levels compared to the normal muscle mass group [37], which resulted in lower TyG index levels. However, these results were based on studies investigating TyG index at a single time point, which may not reflect long-term exposure. With the results of this study, more prospective longitudinal studies are required to explore the long-term relations of the TyG index trajectories and physical function.

The elevated TyG index is associated with impaired β-cell function regardless of the glucose metabolic status [38]. Therefore, TyG index closely mirrors the status of insulin sensitivity. The biological mechanisms underlying the association between the trajectory of the TyG index and frailty have not been clarified to date. Skeletal muscle is the principal place where the insulin regulates glucose uptake [39]. The age-related loss of muscle mass has harmful effects on peripheral glucose absorption through reduced muscle mass for insulin-stimulated glucose disposal, which further leads to hyperinsulinemia status and insulin resistance [40]. Meanwhile, declining physical activity in later life is regarded as one of the main influential factors in insulin resistance [41]. Age-associated metabolic and structural alterations involving chronic inflammation, glycogen synthesis and oxidative pathways also contribute to the malfunction of insulin regulation [42]. Interactively, poor insulin resistance weakens the protein breakdown of skeletal muscle, impairs muscle catabolism and quality, the ensuing sarcopenia is one of the main components of frailty [43, 44]. As a result, the vicious circle of insulin resistance and low muscle mass leads to impaired body energy regulation and physical performance, and subsequently increases the risk of frailty.

In the subgroup analysis within various BMI groups, the association between the high-stable trajectory of the TyG index and frailty risk are prominent in participants with BMI ≥ 24 kg/m^2^. The result was generally consistent with those of other studies [20]. Kim et al. claimed that TyG Index is a potential indicator of sarcopenic obesity in older people [45]. Obese individuals run the risk of experiencing oxidative stress and chronic inflammation, both of which affect the TyG index levels in ways including glucose absorption and adipokine secretion [46]. A common syndrome of “sarcopenic obesity” has been proposed for that these individuals are at an increased risk of adverse events compared to those with obesity or sarcopenia alone [45]. There is evidence that insulin resistance and sarcopenic obesity are closely inter-related. Intramuscular fat infiltration causes insulin resistance, while obesity-induced insulin resistance further facilitates the aging-related decrease of muscle mass. Other than BMI, gender, age and history of hypertension/diabetes did not show any interaction with TyG index. It is worth noting that parts of the association in the subgroup analysis could not reach a significant level. We cannot exclude that the relatively small sample size in the subgroup is a potential Influencing factor. Future research on a larger number of subjects with different clinical characteristics would be helpful in determining the association between TyG index and frailty risk. Sensitivity analyses were conducted to confirm the robustness of the findings. In the analysis restricted to participants without using lipid-lowing medication and hypoglycemic agents, the population who follow the high-stable trajectory of the TyG index bear the highest risk of frailty.

This is the first study to observe a relationship between the TyG index and frailty through the use of frailty index with a ten-year follow-up. The GBTM statistical tool was used to investigate the heterogeneity of the population in the longitudinal observation of the TyG index, which enables us to obtain a reliable trajectory classification. Compared with the single point of information, long-term trajectories of TyG index measurements provided more robust and reliable results. The annual routine health assessment of older urban residents also ensured a consistent assessment of TyG index variability. Furthermore, subgroup analysis and sensitivity analysis were performed to enhance credibility and validity. However, our study had several limitations. Firstly, the current study could not include insulin resistance index values, such as HOMA-IR, as it is not suitable for a large-scale epidemiological survey. However, the TyG index was considered a reliable surrogate marker of insulin resistance and has been shown to be superior to the HOMA-IR for the identification of several insulin-resistance-related conditions [15]. Secondly, in the prospective analysis, the measurement of frailty status was substituted by ADL/IADL due to a lack of data, which might impact the results. Thirdly, we cannot rule out the possibility that residual confounding factors could have biased the results. However, we applied progressive degrees of adjustment for potential confounders. Lastly, the older residents in our study were all from the downtown Fuzhou City, so extrapolations from our results to other populations require further variations.

Long-term elevated levels of TyG index are independently associated with an increased risk of frailty. High levels of TyG index could alert older residents to make early lifestyle changes that may reduce the risk of the progression or incidence of frailty. Relative vigorous control of traditional cardiovascular-metabolic risk factors would be highly beneficial for physical function management. The TyG index could be an alternative surrogate for identifying those elders who are at a high risk of low muscle mass, especially for residents with higher BMI. As a result, it is essential to maintain appropriate levels of TG and FBG within the desirable range and take better control of the long-term TyG index in late life.

## Conclusions

In conclusion, this current study found that older urban residents with long-term elevated TyG index levels have a higher risk of frailty. These findings also highlight the importance of monitoring serum glucose and lipids to prevent the decline of physical capacity for older populations.

### Electronic supplementary material

Below is the link to the electronic supplementary material.

Supplementary Material 1

## Data Availability

The datasets analysed are available upon reasonable request and with permission of the corresponding authors and Fujian Provincial Hospital.

## Abbreviations

TyG index: Triglyceride-glucose index
IR: Insulin resistance
OR: Odds ratio
CI: Confidence interval
GBTM: Group-based trajectory model
MNA-SF: Mini nutritional assessment-short form
ADL: Basic activities of daily living
IADL: Instrumental activities of daily living
