# Supplementary material for: Association of triglyceride-glucose index trajectory and frailty in urban older residents: evidence from the 10-year follow-up in a cohort study
Source: Cardiovasc Diabetol. 2023 Sep 29;22:264. doi: 10.1186/s12933-023-02002-3 (PMC10542691; doi:10.1186/s12933-023-02002-3)
Supplement: Supplementary file 1 — Supplementary Material 1 [file 12933_2023_2002_MOESM1_ESM.docx]

**Association of Triglyceride-glucose index trajectory and frailty** **in urban older residents: evidence from 10-year follow-up in a cohort study**

Yin Yuan^#^, Simiao Chen^#^, Chunjin Lin^#^, Xiaoming Huang, Siyang Lin, Feng Huang*, and Pengli Zhu*

**Additional file 1**

**Contents:**

**Supplementary table 1** List of 40 variables included in the frailty index

**Supplementary table 2** The statistics for trajectory models for TyG index from 2011-2020

**Supplementary table 3** The Maximum Likelihood Estimates from the Censored Normal Model

**Supplementary table 4** Association of TyG index and frailty index in 2020

**Supplementary table 5** Sensitivity Analysis for the association of TyG trajectories and frailty risk

**Supplementary table 6** Subgroup analysis for the prefrailty and frailty risk according to trajectories of TyG index from 2011 to 2020

**Supplementary table 7** Association of TyG index with one-year ADL/IADL decline (2020-2021)

**Supplementary** **table 1 List of 40 variables included in the frailty index**

| **Category** | **Variables** | **Definition** |
| --- | --- | --- |
| Basic/instrumental activities of daily living (ADL/IADL) | Bathing | Independence = 0, Need help = 1 |
|  | Dressing | Independence = 0, Need help = 1 |
|  | Toileting | Independence = 0, Need help = 1 |
|  | Transferring | Independence = 0, Need help = 1 |
|  | Continence | Independence = 0, Need help = 1 |
|  | Feeding | Independence = 0, Need help = 1 |
|  | Use Telephone | Independence = 0, Need help = 1 |
|  | Shopping | Independence = 0, Need help = 1 |
|  | Food Preparation | Independence = 0, Need help = 1 |
|  | Housekeeping | Independence = 0, Need help = 1 |
|  | Laundry | Independence = 0, Need help = 1 |
|  | Transportation | Independence = 0, Need help = 1 |
|  | Responsibility for own medications | Independence = 0, Need help = 1 |
|  | Handle Finances | Independence = 0, Need help = 1 |
| Physical health and medical conditions | Self rating of health | Poor = 1, Fair = 0.75, Good = 0.5, V. Good = 0.25, Excellent = 0 |
|  | Hypertension | No = 0, Yes = 1 |
|  | Diabetes | No = 0, Prediabetes = 0.5, Yes = 1 |
|  | Coronary heart disease | No = 0, Yes = 1 |
|  | Heart failure | No = 0, Yes = 1 |
|  | Cancer | No = 0, Yes = 1 |
|  | Stroke | No = 0, Yes = 1 |
|  | Chronic respiratory disease | No = 0, Yes = 1 |
|  | Chronic kidney diseases | No = 0, Yes = 1 |
| Geriatric syndrome | Polypharmacy | No = 0, Yes = 1 |
|  | Malnutrition | MNA-SF score ≥12 = 0, <12 = 1 |
|  | Exercise (expenditure of physical activity per week < 383 kcal for men, < 270 kcal for women) | Meet the standard = 0, not = 1 |
|  | Vision | Normal = 0, mild impaired = 0.5, impaired = 1 |
|  | Hearing | Normal = 0, mild impaired = 0.5, impaired = 1 |
|  | Persistent fatigue | No = 0, Yes = 1 |
|  | Balance | TUG <10s =0,10~20s=0.5, >20s=1 |
|  | Incontinence | No = 0, Occasionally = 0.5, Yes = 1 |
|  | History of fall | No = 0, one time = 0.5, ≥ 2 times or severely injured = 1 |
|  | Anxiety | “GAD-7≤ 4” = 0, “GAD-7 5~13” = 0.5, “GAD-7≥14” = 1 |
|  | Depression | “GDS-4=0”=0, “GDS-4=1” = 0.5, “GDS-4≥2” =1 |
|  | Sleep | “AIS<4” = 0, “AIS 4~5” = 0.5, “AIS ≥ 6” = 1 |
|  | Chronic pain | No = 0, Yes = 1 |
|  | Cognition | “Minicog >3” =0, “Minicog =3” =0.5, “Minicog ≤2” =1 |
|  | Grip strength | Fried’s standard^[1]^ |
|  | Gait speed | Fried’s standard^[1]^ |
|  | Family support | SSRS ≥11=0, <11 =1 |

MNA-SF: mini nutritional assessment-short form, GAD-7: generalized anxiety disorder scale-7, GDS-4: geriatric depression scale-4, AIS: Athens insomnia scale, TUG: timed up and go test, SSRS: social support rating scale.

[1] Fried L P, Tangen C M, Walston J, et al. Frailty in older adults: evidence for a phenotype. J Gerontol A Biol Sci Med Sci. 2001,56(3):M146-M156. DOI: 10.1093/gerona/56.3.m146.

**Supplementary table 2 The statistics for trajectory models for TyG index from 2011-2020**

| **Trajectory groups** | **Trajectory shape parameter ^1^** | **BIC**  **n=7635** | **BIC**  **n=7635** | **AIC**  **n=7635** | **Group membership**  **(%)** | **Group Avepp** | **Occ** |
| --- | --- | --- | --- | --- | --- | --- | --- |
| 2 | (3 3) | -5659.43 | -5652.26 | -5624.72 | 0.641 (a) | 0.912 (b) | 5.80 |
|  |  |  |  |  | 0.359 | 0.880 | 13.09 |
| 2 | (2 2) | -5656.29 | -5650.55 | -5628.53 | 0.643 | 0.913 | 5.83 |
|  |  |  |  |  | 0.357 | 0.879 | 13.08 |
| 3 | (2 2 2) | -5192.41 | -5183.81 | -5150.77 | 0.384 | 0.862 | 10.02 |
|  |  |  |  |  | 0.499 | 0.845 | 5.47 |
|  |  |  |  |  | 0.117 | 0.879 | 54.82 |
| 3 | (3 2 2) | -5186.42 | -5177.11 | -5141.31 | 0.383 | 0.858 | 9.73 |
|  |  |  |  |  | 0.500 | 0.845 | 5.45 |
|  |  |  |  |  | 0.117 | 0.882 | 56.41 |
| 4 | (2 2 2 2) | -5063.24 | -5051.78 | -5007.72 | 0.131 | 0.813 | 28.84 |
|  |  |  |  |  | 0.427 | 0.769 | 4.47 |
|  |  |  |  |  | 0.360 | 0.819 | 8.04 |
|  |  |  |  |  | 0.081 | 0.904 | 106.84 |

BIC: Bayesian information criterion, AIC: Akaike information criterion, Avepp: Average posterior probability, Occ: Odds of correct classification.

^1^ Defines the shape parameters of the trajectory groups: 0 = intercept only, 1 = linear, 2 = quadratic, 3 = cubic.

**Supplementary table 3 The Maximum Likelihood Estimates from the Censored Normal Model**

| **Group** | **Parameter** | **Estimate** | **Standard Error** | **T for H0: Parameter=0** | **Prob > \|T\|** |
| --- | --- | --- | --- | --- | --- |
| 1 | Intercept | 8.71580 | 0.04118 | 211.650 | <0.001 |
|  | Linear | -0.25678 | 0.03592 | -7.150 | <0.001 |
|  | Quadratic | 0.04588 | 0.00841 | 5.453 | <0.001 |
|  | Cubic | -0.00254 | 0.00056 | -4.546 | <0.001 |
|  |  |  |  |  |  |
| 2 | Intercept | 9.12707 | 0.02440 | 374.120 | <0.0001 |
|  | Linear | -0.10314 | 0.01128 | -9.147 | <0.0001 |
|  | Quadratic | 0.00863 | 0.00115 | 7.491 | <0.0001 |
|  |  |  |  |  |  |
| 3 | Intercept | 9.88731 | 0.04713 | 209.802 | <0.001 |
|  | Linear | -0.08819 | 0.02177 | -4.051 | 0.0001 |
|  | Quadratic | 0.00463 | 0.00220 | 2.107 | 0.0351 |
|  |  |  |  |  |  |
|  | Sigma | 0.40636 | 0.00352 | 115.307 | <0.001 |
|  |  |  |  |  |  |
| **Group membership** | |  |  |  |  |
|  | Group 1 (%) | 38.29194 | 1.97521 | 19.386 | <0.001 |
|  | Group 2 (%) | 49.96318 | 1.88362 | 26.525 | <0.001 |
|  | Group 3 (%) | 11.74488 | 0.98293 | 11.949 | <0.001 |

**Supplementary table 4 Association of TyG index and frailty index in 2020**

| **Variables** | **Coefficient β (95% CI)** | ***P* value** |
| --- | --- | --- |
| TyG index | 0.014 (0.007, 0.020) | **<0.001** |
| Age | 0.004 (0.003, 0.004) | **<0.001** |
| BMI | 0.005 (0.004, 0.006) | **<0.001** |
| MNA-SF score | -0.021(-0.023, -0.019) | **<0.001** |
| HbA1c | 0.006 (0.003, 0.009) | **<0.001** |
| TC | -0.007 (-0.010, -0.004) | **<0.001** |
| _cons | -0.119 (-0.186, -0.052) | **<0.001** |

MNA-SF: mini nutritional assessment-short form. The model was adjusted for age, BMI, MNA-SF score, LDL-C, TC, HDL-C, HbA1c, MAP.

**Supplementary table 5 Sensitivity Analysis for the association of TyG trajectories and frailty risk**

| TyG index  trajectories | Model 1  OR (95% CI) | *P* value | Model 2  OR (95% CI) | *P* value |
| --- | --- | --- | --- | --- |
| **Excluding participants with the use of lipid-lowering medication (n=504)** | | | | |
| **Prefrailty risk** |  |  |  |  |
| Low-stable | Reference |  | Reference |  |
| Moderate-stable | 1.39 (1.06~1.82) | **0.016** | 0.94 (0.70~1.26) | 0.170 |
| High-stable | 2.19 (1.35~3.56) | **0.002** | 1.63 (1.01~2.79) | **0.046** |
| **Frailty risk** |  |  |  |  |
| Low-stable | Reference |  | Reference |  |
| Moderate-stable | 1.94 (1.09 ~3.46) | **0.025** | 1.55 (0.84~2.87) | 0.110 |
| High-stable | 6.24 (2.62~14.89) | **<0.001** | 3.69 (1.34~10.13) | **0.011** |
| **Excluding participants with the use of hypoglycemic agents (n=423)** | | | | |
| **Prefrailty risk** |  |  |  |  |
| Low-stable | Reference |  | Reference |  |
| Moderate-stable | 1.14 (0.89~1.47) | 0.295 | 1.27 (0.96~1.68) | 0.092 |
| High-stable | 1.46 (0.92~2.34) | 0.112 | 1.60 (0.95~2.72) | 0.080 |
| **Frailty risk** |  |  |  |  |
| Low-stable | Reference |  | Reference |  |
| Moderate-stable | 1.11 (0.66~1.88) | 0.683 | 1.31 (0.72~2.37) | 0.380 |
| High-stable | 2.44 (1.02~5.88) | **0.046** | 2.64 (1.06~4.61) | **0.041** |

Model 1: Adjusted for age, gender, smoking, drinking, BMI, nutritional status, and exercise; Model 2: adjusted for model 1 covariates plus chronic disease history, TC, LDL-C, HDL-C, MAP, HbA1c, hypoglycemic agents, and lipids-lowering medication.

**Supplementary table 6 Subgroup analysis for the prefrailty and frailty risk according to trajectories of TyG index from 2011 to 2020**

|  | No. of participants | Prefrailty | | | Frailty | | |
| --- | --- | --- | --- | --- | --- | --- | --- |
|  |  | Moderate-stable | High-stable | *P* for interaction | Moderate-stable | High-stable | *P* for interaction |
| Age (yrs) |  |  |  |  |  |  |  |
| <70 | 815 | 1.03 (0.71, 1.50) | 1.65 (0.87, 3.15) | 0.225 | 1.76 (0.57, 5.42) | 2.11 (0.40, 11.16) | 0.799 |
| ≥70 | 1006 | **1.59 (1.06, 2.38)** | **2.19 (1.07, 4.47)** |  | 1.33 (0.73, 2.41) | **2.81 (1.07, 7.36)** |  |
| Gender |  |  |  |  |  |  |  |
| Male | 745 | 1.10 (0.73, 1.65) | 1.68 (0.84, 3.38) | 0.447 | 0.89 (0.34, 2.36) | 2.13 (0.54, 8.36) | 0.202 |
| Female | 1076 | 1.25 (0.87, 1.81) | 1.77 (0.91, 3.45) |  | 1.27 (0.69, 2.36) | 2.22 (0.81, 6.09) |  |
| BMI |  |  |  |  |  |  |  |
| <24kg/m^2^ | 751 | 1.25 (0.82, 1.89) | 1.74 (0.76, 3.99) | **0.041** | 2.41 (0.99, 5.38) | 2.30 (0.53, 10.02) | **0.044** |
| ≥ 24kg/m^2^ | 1070 | 1.12 (0.78, 1.61) | **1.87 (1.03, 3.41)** |  | 0.61 (0.31, 1.20) | **2.11 (1.05, 3.24)** |  |
| History of diabetes | |  |  |  |  |  |  |
| Yes | 587 | 0.91 (0.42, 1.95) | 2.08 (0.73, 5.94) | 0.072 | 0.57 (0.21, 1.55) | 1.50 (0.40, 5.58) | 0.273 |
| No | 1234 | 1.17 (0.87, 1.58) | 1.25 (0.69, 2.25) |  | 1.31 (0.67, 1.58) | 1.78 (0.45, 7.09) |  |
| History of hypertension | |  |  |  |  |  |  |
| Yes | 1129 | 0.72 (0.47, 1.09) | 1.16 (0.59, 2.26) | 0.348 | 0.61 (0.32, 1.17) | 1.40 (0.53, 3.68) | 0.165 |
| No | 692 | **1.50 (1.00, 2.26)** | **2.21 (1.03, 4.73)** |  | 2.18 (0.81, 5.91) | 1.93 (0.26, 14.47) |  |

**Supplementary table 7 Association of TyG index with one-year ADL/IADL decline (2020-2021)**

|  | TyG index quartiles (N=1292) | | | |
| --- | --- | --- | --- | --- |
|  | Q1 [7.25-8.53] | Q2 [8.53-8.88] | Q3 [8.88-9.24] | Q4 [9.24-10.9] |
| Case/Total | 11/292 | 21/284 | 18/299 | 56/417 |
| ORs (95% CI) | Reference | 2.12 (0.97~4.63) | 1.63 (0.72~3.69) | 2.09 (1.02~4.45) |
| *P* value | - | 0.06 | 0.237 | **0.045** |

The model was adjusted for age, gender, smoking, drinking, BMI, nutritional status, chronic disease history (has any of the following medical conditions: hypertension, diabetes, dyslipidemia, cerebrovascular/cardiovascular disease), TC, HbA1c, mean arterial pressure, and polypharmacy.
